# Altered brain metabolites in patients with diabetes mellitus and related complications – evidence from ^1^H MRS study

**DOI:** 10.1042/BSR20180660

**Published:** 2018-09-07

**Authors:** Xue Zhao, Qing Han, Xiaokun Gang, Guixia Wang

**Affiliations:** 1 Department of Endocrinology and Metabolism, The First Hospital of Jilin University, Changchun 130021, Jilin Province, China; 2 Department of Orthopedics, The Second Hospital of Jilin University, Changchun 130021, Jilin Province, China

**Keywords:** biomarkers, metabolite, NMR spectroscopy, neuroimaging, type 2 diabetes

## Abstract

In recent years, diabetes mellitus (DM) has been acknowledged as an important factor for brain disorders. Significant alterations in brain metabolism have been demonstrated during the development of DM and its complications. Magnetic resonance spectroscopy (MRS), a cutting-edge technique used in biochemical analyses, non-invasively provides insights into altered brain metabolite levels *in vivo*. This review aims to discuss current MRS data describing brain metabolite levels in DM patients with or without complications. Cerebral metabolites including *N*-acetylaspartate (NAA), creatine (Cr), choline (Cho), *myo*-inositol (mI), glutamate, and glutamine were significantly altered in DM patients, suggesting that energy metabolism, neurotransmission, and lipid membrane metabolism might be disturbed during the progression of DM. Changes in brain metabolites may be non-invasive biomarkers for DM and DM-related complications. Different brain regions presented distinct metabolic signatures, indicating region-specific diabetic brain damages. In addition to serving as biomarkers, MRS data on brain metabolites can also shed light on diabetic treatment monitoring. For example, exercise may restore altered brain metabolite levels and has beneficial effects on cognition in DM patients. Future studies should validate the above findings in larger populations and uncover the mechanisms of DM-induced brain damages.

## Introduction

Diabetes mellitus (DM) is a global burden with dramatic morbidity and mortality rates [1,2]. In 2013, the International Diabetes Federation (IDF) presented that the global DM incidence rate and impaired glucose tolerance (IGT) rate was 6.9 and 8.3%, respectively [3]. It was predicted that prevalence of DM and the IGT would reach 8.0 and 10.1% by 2035 [3,4]. In addition to the striking diabetic epidemiology data, DM contributes to numerous complications, such as diabetic retinopathy (DR), diabetic kidney disease (DKD), diabetic peripheral neuropathy (DPN), and diabetic cardiomyopathy (DCM) [5–8]. Poor glycemic control coupled with long DM duration induces rapid deterioration of these comorbidities and increases DM-induced mortality rate.

In recent years, emerging evidences have shown that DM affects brain metabolism during its progression, ultimately leads to DM-related metabolic disorders in central nervous system (CNS) [9–12]. The term ‘diabetic encephalopathy’ denotes an intimate relationship between DM and brain dysfunction, especially for aging, Alzheimer’s disease (AD), and depression [13–15]. However, the specific mechanism of DM-induced brain metabolic alterations was unclear. Studies have shown DM can induce significant differences in brain metabolite levels in both animal models and human subjects [4,10,16–18]. Thus, exploring the brain metabolite levels in different stages of DM during its progression may help to determine how DM mechanistically contributes to brain disorders, such as AD and depression. However, available reviews focussing on brain metabolites in patients with diabetes is limited.

Magnetic resonance spectroscopy (MRS), a cutting-edge technique of biochemical analyses, detects the alterations in metabolic neurochemical levels and energetics in different regions of the brain *in vivo* [10,19–22]. Given that MRS is both safe and non-invasive, researchers have applied MRS (especially ^1^H-MRS) to investigate the underpins of many neurological conditions, such as AD and DM [23–25]. To date, over 20 brain metabolites have been detected using high-filed MRI/MRS systems in animal models. Those metabolites include, lactate (Lac), alanine (Ala), γ-Aminobutyric acid (GABA), *N*-acetylaspartate (NAA), *N*-acetylaspartylglutamate (NAAG), glutamate (Glu), glutamine (Gln) glutathione (GSH) aspartate (Asp), phosphocreatine (PCr), creatine (Cr), phosphrylcholine (PC), glycerylphosphorylcholine, taurine (Tau), *scyllo*-inositol, *myo*-inositol (mI), glycine, ascorbate (Asc), glucose, and phosphorylethanolamine [10,19,20]. Monitoring changes in each metabolite level and ratios of metabolites could provide information about neuronal damages, membrane metabolism dysfunctions and neurotransmission defects occurring in neurological diseases. Additionally, the MRS platform can reflect the influences of neurological conditions on metabolic pathways in specific parts of the brain.

The present review summarizes available data on brain metabolite levels in patients with DM and DM-related comorbid complications, in order to identify non-invasive biomarkers for diabetes-induced brain metabolic changes during DM progression.

## Brain metabolite alterations in type 1 and type 2 DM patients

DM is classified into type 1 diabetes (T1DM), type 2 diabetes (T2DM), gestational DM (GDM), and other specific types of diabetes, based on underlying disease mechanisms and the demand for insulin. In all diabetic patients, T1DM and T2DM patients make up a vast majority. Detailed characteristics of brain metabolite patterns identified by ^1^H-MRS in patients with T1DM or T2DM are given in Table 1.

**Table 1:** Clinical parameters related to brain metabolite levels in T1DM and T2DM patients

Study	Year	Number	Age (years)	DM duration (years)	HbA1c (%)	Testing area	Magnetic parameters	Identified brain metabolites
Sinha et al. [31]	2014	Control: 7 T2DM: 10	Control: 27.6 ± 2.8 T2DM: 39 ± 5.2	3.5 ± 1.2	7.25 (6.8, 8.9)	Right frontal, right parieto-temporal, right parieto-occpital white matter	Strength: 1.5 T TR: 2000 ms TE: 30 ms	NAA; Cho; Cr; Glu; Gln; mI; glucose; Cr + PCr; Glu + Gln
Lin et al. [32]	2013	Control: 22 T2DM: 20	Control: 52–80 T2DM: 51–85	N/A	5.8–14.3	Lenticular nuclei; thalamus	Strength: 1.5 T TR: 1000 ms TE: 144 ms	NAA/Cr; Cho/Cr; lactic acid
Mangia et al. [26]	2013	Control: 32 T1DM: 13	Control: 36 ± 10 T1DM: 41 ± 11	22 ± 12	7.5 ± 2	Occipital lobe (grey matter); parieto-occpital region (white matter)	Strength: 4 T TR: 4.5 s TE: 4 ms	Ala; Asp; Asc; GPC + PC; Cr; PCr; GABA; Glc; Gln; Glu; GSH; *myo*-Ins; *scyllo*-Ins; Lac; NAA; NAAG; PE; Tau
Heikkilä et al. [27]	2010	Control: 11 T1DM: 7	Control: 30.8 ± 6.9 T1DM: 29.4 ± 4.5	12.4 ± 2.8	7.6 ± 0.8	Frontal cortex; thalamus; white matter	Strength: 1.5 T TR: 3000 ms TE: 30 ms	NAA; tCr; glucose
Heikkilä et al. [28]	2009	Control: 12 T1DM: 17	Control: 29.5 ± 10 T1DM: 28.2 ± 4.4	7 ± 5	7.4 ± 1.1	Frontal cortex; thalamus; white matter	Strength: 1.5 T TR: 3000 ms TE: 30 ms	NAA; Cr; Cho; mI; glucose
Sahin et al. [16]	2008	Control: 14 IGT: 13 T2DM: 25	Control: 41.5 ± 8.1 IGT: 44.5 ± 11.3 T2DM: 45.6 ± 14.1	N/A	control: 7.9 ± 1.17 DM: 13.6 ± 1.5	Frontal cortex; thalamus; parietal white matter	Strength: 1.5 T TR: 2000 ms TE: 31 ms	NAA/Cr; Cho/Cr; mI/Cr
Seaquist et al. [29]	2005	Control: 14 T1DM: 8 T2DM: 6	Control: 37 ± 9 DM: 43 ± 13	N/A	9.8 ± 1.7	Occipital cortex	Strength: 4.0 T TR: 4.5 ms TE: 4–20 ms	Glc; Ins; Cho; Glu; NAA
Sarac et al. [30]	2005	Control: 14 T1DM: 30	Control: 13.1 ± 3.5 T1DM: 12.1 ± 3.3	N/A	11.9 ± 3.4	Pons, left basal ganglion; left posterior parietal white matter	Strength: 1.5 T TR: 2000 ms TE: 136 ms	NAA; Cho; Cr; NAA/Cr; Cho/Cr

Abbreviations: Cho, choline; HbA1c, glycosylated hemoglobin A1c; ms, millisecond; N/A, none; PE, phosphoethanolamine; tCr, total Cr; TE, echo time; TR, repetition time.

### T1DM

Amongst studies focussing on T1DM, the majority of patients were adults ranging from 25 to 45 years [26–29]. Only one study focussed on children with a mean age of 12–13 years [30]. Mangia et al. [26] performed hyperglycemic clamp tests on 13 patients with well-controlled T1DM (HbA_1_C (glycosylated hemoglobin): 7.5 ± 2%; DM duration: 22 ± 12 years) and 32 matched healthy controls. The results revealed that NAA and Glu were reduced by 6% (*P*<0.05) in the grey matter (occipital lobe) of T1DM patients. No significant differences were identified in other brain metabolite levels. A similar decrease in NAA (6%) was also demonstrated by Heikkilä et al. [28]. In that study, brain metabolite and glucose levels were detected in the frontal cortex, white matter and thalamic regions of 17 T1DM patients and 12 healthy controls. NAA and mI levels were elevated by 20% in the frontal white matter (*P*<0.001), and by 8% in frontal cortex (*P*=0.042) of T1DM patients. Moreover, brain glucose levels positively correlated with fasting plasma glucose (r = 0.88; *P*<0.01), indicating peripheral glucoses were important determinants of brain energy expenditures. To explore affects of hyperglycemia on brain glucose levels, Heikkilä et al. [27] performed a 2-h hyperglycemic clamp test with glucose increase of 12 mmol/l. Significant decrease in glucose uptake was observed in thalamic region when compared with cortex and white matter regions (*P*=0.011), suggesting the involvement of thalamic region in the progression of hyperglycemia-induced brain disorders. Seaquist et al. [29] found that patients with poor glycemic control (HbA_1_C: 9.8 ± 1.7%) presented nearly 10% lower in brain glucose level in occipital cortex compared with healthy controls. These results demonstrated the influence of chronic hyperglycemia on brain glucose level as well as the presentation of distinct metabolite signatures. Moreover, the impaired glucose uptake may explain neurone loss and functional impairment comorbidities that occurred in elderly DM patients. Another study by Sarac et al. [30] revealed that NAA/Cr and Cho (Choline)/Cr ratios in the pons and NAA/Cr ratio in the left posterior parietal white matter region were reduced in children with T1DM (13.1 ± 3.5 years old) compared with healthy controls. Taken together, reduced NAA level was found both in T1DM adults and children. However, these results require confirmation in larger cohorts across ethnic backgrounds.

### T2DM

Global incidence of T2DM has grown dramatically in the past decades. Several studies have reported significant brain metabolic alterations in patients with T2DM. Sinha et al. [31] showed that NAA level was significantly decreased in the right frontal and parieto-temporal region of ten patients with T2DM (*P*=0.03), while Glx level (Glu + Gln) was increased in the right frontal region (*P*=0.01) when compared with healthy controls. Consistent with the results presented above in T1DM, brain glucose levels were increased in all regions of the brain in patients with T2DM. Lin et al. [32] detected NAA, Cho, Cr levels in lenticular nuclei and thalamic regions. The results showed that NAA/Cr ratio was decreased (*P*=0.007) and Cho/Cr ratio was increased (*P*=0.001) in patients with T2DM. Additionally, NAA/Cr ratio was negatively correlated with fasting blood glucose (r_1_ = −0.573) and HbA_1_C levels (r_2_= −0.564). Conversely, Cho/Cr ratio was positively correlated with the same variables. Thus, the DM-induced changes in brain metabolite levels were identified across extensive brain regions and these changes were associated with the peripheral glucose metabolism. However, it is still uncertain when these changes occur in the disease course and whether these changes happen before the diagnosis of DM. Sahin et al. [16] investigated brain metabolites in patients with IGT (*n*=13) and T2DM (*n*=25). The results showed that in the frontal cortex region, IGT patients had higher Cho/Cr when compared with healthy controls (*P*=0.038). However, in parietal white matter, Cho/Cr was significantly lower in T2DM patients than in controls (*P*=0.049). Patients with poorly controlled T2DM (HbA_1_C >10%) presented lower Cho/Cr ratio than patients with well-controlled T2DM patients (HbA_1_C < 10%; *P*=0.012). Based on these collective findings, brain metabolites differed amongst healthy controls and patients experiencing either IGT or T2DM patients. These findings provide evidence of specific MRS landmarks that indicate altered brain metabolism during DM progression. Future studies should focus on the primary changes in diabetic brain metabolism and identify the key changes to brain metabolites induced by hyperglycemia.

## Brain metabolite alterations in diabetic patients with chronic comorbid complications

Chronic hyperglycemia is an important risk factor for organ damages or dysfunctions in DM patients. The most frequently affected organs are kidney, eyes, and nervous system [33], namely, DKD, retinopathy, and neuropathy, respectively. Studies have shown significant changes in brain metabolite in DM comorbid complications. Details of study characteristics and identified brain metabolites are presented in Table 2.

**Table 2:** Clinical parameters of studies focussing on brain metabolite levels in diabetic patients with chronic complications (DR, DKD, DPN and DR+DN)

Study	Year	Number	Age (years)	DM duration (years)	HbA1c (%)	Testing area	Magnetic parameters	Identified brain metabolites
**DR**								
Tong et al. [37]	2014	Control: 30 T2DM: 30 DR: 29	Control: 51.4 ± 5.3; T2DM: 51.2 ± 5; DR: 51.2 ± 5.7	N/A	T2DM: 8.2 ± 1.7; DR: 8.2 ± 1.5	Left frontal white matter, lenticular nucleus; optic radiation	Strength: 3.0 T TR: 2000 ms TE: 30 ms	NAA; Cho; Cr; mI; NAA/Cr; mI/Cr;NAA/Cho; Cho/Cr
Ozsoy et al. [17]	2012	Control: 15 DM: 15 NPDR: 15 PDR: 15	Control: 51 ± 7.4 DM: 50.8 ± 7.2 NPDR: 54.8 ± 8.4 PDR: 56.5 ± 8.3	N/A	N/A	Left visual cortex	Strength: 1.5 T TR: N/A TE: N/A	NAA; Cho; Cr; NAA/Cr; NAA/Cho; Cho/Cr
**DKD**								
Shi et al. [46]	2015	NC: 30 DM: 33 DN-III: 26 DN-IV: 27	NC: 55.8 ± 9.2 DM: 53.6 ± 11.2 DN-III: 56.9 ± 9.1 DN-IV: 57.3 ± 8.8	N/A	DM: 9.1 ± 2.5 DN-III: 9.6 ± 2.9 DN-IV: 8.9 ± 2.3	Basal ganglia; hippocampus	Strength: 1.5 T TR: N/A TE: N/A	NAA; Cr; Cho; NAA/Cr; Cho/Cr
Fiorina et al. [45]	2012	NC: 8 ESRD: 15 KD: 9 KP: 14	NC: 42 ± 5 ESRD: 48.7 ± 7.3 KD: 46 ± 6.5 KP: 43.2 ± 5.5	ESRD: 31.6 ± 8.2 KD: 35.8 ± 6.6 KP: 30.1 ± 6.8	ESRD: 7.9 ± 1.5 KD: 7.9 ± 1.3 KP: 5.8 ± 0.9	Bifrontal cortex	Strength: 1.5 T TR: 2600 ms TE: 135 ms	NAA; Cho; Cr; NAA/Cho; NAA/Cr; Cho/Cr
**DPN**								
Selvarajah et al. [43]	2008	Control: 6 DM: 8 DPN: 10	Control: 42.5 ± 15.4 DM: 42.9 ± 8.7 DPN: 48.3 ± 11.5	DM: 11.6 ± 7.7 DPN: 22.1 ± 8.8	DM: 7.8 ± 0.9 DPN: 9.1 ± 1.2	Thalamus	Strength: 1.5 T TR: 5000 ms TE: 20 ms	NAA; Cho; Cr; Glx; Lac; mI; NAA/Cr; NAA/Cho; Cho/Cr
Sorensen et al. [42]	2008	Control: 6 DM: 14 DN: 12	Control: 48 (33, 60) DM: 57 (53, 63) DN: 61 (56, 68)	DM: 13.5 (8.8, 27) DN: 15 (10.3, 22.8)	DM: 7.7 (6.7, 8.9) DN: 7.5 (6.8, 8.6)	Left thalamus; anterior cingulate cortex; dorsolateral prefrontal cortex	Strength: 1.5 T TR: 1500 ms TE: 25 ms	NAA; Cr; Cho
**DR+DN**								
Mäkimattila et al. [38]	2004	Control: 10 DR + DN: 10	Control: 36.2 ± 4.1; DR and DN: 36.2 ± 3.3	27.8 ± 3.1	9.1 ± 0.9	Frontal cortex; thalamus; posterior frontal white matter	Strength: 1.5 T TR: 3000 ms TE: 35 ms	NAA; Cho; mI; glucose

Abbreviations: DN, diabetic neuropathy; ESRD, end-stage renal disease; Ins, inositol; KD, kidney transplantation; KP, kidney-pancreas transplantation; N/A, none; NC, normal control; NPDR: non-proliferative DR; PDR, proliferative DR; TE, echo time; TR, repetition time.

### DR

DR is a major cause of blindness in working-age adults around the world. DR is usually divided into two major stages: non-proliferative DR (NPDR) and proliferative DR (PDR) [34]. Previous studies reported a close relationship between retinopathy and both cerebral damage and stroke [35,36]. A study identifying the differences in brain metabolite levels amongst DR patients, DM patients and healthy controls revealed that NAA/Cr ratio in the frontal white matter (*P*=0.011) and optic radiation regions (*P*=0.037) were significantly lower in DR patients when compared with DM patients [37]. The mI/Cr ratio showed a 22% increase in DM patients when compared with healthy controls (*P*=0.009). Additionally, mI/Cr ratio was 24.6% lower in DR patients when compared with DM patients (*P*=0.001). The correlative analysis showed that NAA/Cr ratio was inversely correlated with DR severity (r = −0.511; *P*=0.005). Thus, decreases in NAA/Cr and mI/Cr ratios in DR patients may be associated with DR progression. Another study by Ozsoy et al. [17] in patients with NPDR and PDR showed that NAA/Cr and Cho/Cr ratios in the left visual cortex region were significantly different in patients with HbA1C > 8% and HbA1C < 8% (*P*<0.05). In addition, NAA/Cr and Cho/Cr were numerically reduced in PDR compared with NPDR patients although no significant difference was observed. Generally, the development of retinopathy was accompanied with other microvascular complications, including kidney disease and neuropathy. Mäkimattila et al. [38] detected the differences of brain metabolites in frontal cortex, thalamic and posterior frontal white matter between ten healthy controls and ten patients with both retinopathy and autonomic neuropathy. They found that Cho and mI levels were increased in the white matter by 21% (*P*=0.02) and 30% (*P*=0.007) in retinopathy and autonomic neuropathy patients, respectively, but NAA level was unaltered. These data suggested that Cho and mI levels might play a role in the progression of complex diabetic complications. Moreover, these variables were related to membrane damage and proliferation or activation of glia cells [39,40], further implicating brain metabolites in the development of diabetic complications.

### DPN

Clinical manifestations of DPN include paresthesia, sensibility loss, ulcers, and osteomyelitis [41]. However, the underlying mechanism of DM neuropathy is unclear. Until recently, little evidence was available to establish a relationship between peripheral neuropathy and altered brain metabolite levels. Sorensen et al. [42] designed a study with three subgroups: T2DM patients with neuropathic pain (*n*=12), T2DM patients without pain (*n*=14), and healthy controls (*n*=18) to explore whether brain metabolite levels changed in patients with diabetic neuropathy. The results showed decreased NAA and Cr levels in dorsolateral prefrontal cortex region and thalamic region in DM group when compared with healthy controls (*P*<0.05). Patients with DPN presented lower NAA in the thalamic region than DM patients without pain. In the thalamic region, Selvarajah et al. [43] found that NAA/Cr and NAA/Cho ratio were lower in DPN patients compared with DM patients and healthy controls (*P*=0.04; *P*=0.02). Moreover, NAA/Cr level positively correlated with sural amplitudes (r = 0.61; *P*=0.004) and nerve conduction velocities (r = 0.58; *P*=0.006). Thus, DM (or high glucose concentrations) could affect both brain and peripheral nerve metabolism. The impact of changes in brain metabolite levels during DPN development should be researched carefully in future studies.

### DKD

The incidence of patients with chronic kidney disease (CKD) has increased in the past 10 years. It has been considered as a great economic burden due to the striking number of patients with end-stage renal disease (ESRD) and increased requirement for renal replacement therapy [44]. Fiorina et al. [45] examined the brain metabolites of ESRD patients (*n*=15), kidney transplantation patients (KD, *n*=9) and kidney-pancreas transplantation patients (KP, n=14) and found decreased NAA/Cho ratio in ESRD, KD, KP groups when compared with healthy controls (*P*<0.05). NAA/Cr ratio was significantly lower in ESRD group than in KD and KP groups (*P*<0.01). These data showed that kidney and kidney-pancreas transplantation normalized the renal function and rescued DM-induced impaired brain metabolism to some extent. To identify the difference in brain metabolite levels in patients experiencing different DKD stages, Shi et al. [46] divided enrolled patients into four groups: DM patients (urinary albumin excretion rates (UAER) <20 μg/min) DN-III patients (20 μg/min ≤ UAER < 200 μg/min), DN-IV patients (UAER ≥ 200 μg/min), and healthy controls. When compared with DM patients, NAA and Cho levels were reduced in DN-III and DN-IV patients (*P*<0.05). Significant differences in NAA and Cho levels were observed between DN-III and DN-IV patients (*P*<0.05). Therefore, the findings support that brain metabolite levels are altered during different stages of DKD. Due to the limited evidence in this field, the underlying mechanism is unclear. But the degree of glucose control, and diabetic stages might be important factors influencing the alterations of metabolic molecules in brain detected by MRS [47,48]. Future studies should discover the mechanism further in a larger population.

## Alterations of brain metabolites in diabetic patients with acute complications

Acute diabetic complications usually occur in T1DM patients, including diabetic ketoacidosis (DKA), hypoglycemia coma, hyperosmolar coma, and lactic acidosis. In the present review, studies were identified that investigated the influence of acute DM complications on brain metabolite levels. The characteristics of this collection of literature are given in Table 3.

**Table 3:** Clinical parameters of studies focussing on brain metabolite levels in diabetic patients with acute complications (hypoglycemia and (DKA)

Study	Year	Number	Age (years)	DM duration (years)	HbA1c (%)	Testing area	Magnetic parameters	Identified brain metabolites
**Hypoglycemia**								
Wiegers et al. [54]	2016	Control: 7 IAH: 7 NAH: 7	Control: 27.6 ± 6.9; IAH: 24.7 ± 8.1; NAH: 26.2 ± 5.8	10 (2.5, 17.5)	IAH: 7.5 ± 0.6 NAH: 7.3 ± 0.4	White matter, grey matter; spinal fluid	Strength: 3.0 T TR: 3000 ms TE: 144 ms	NAA; tCho; tCr; mI; Asp; Glu; Gln; Tau; inositol
**DKA**								
Wootton-Gorges et al. [50]	2007	DKA: 29	11.9 ± 3.0	New onset (45%)	N/A	Occipital grey matter; basal ganglia; periaqueductal grey matter	Strength: 1.5 T TR/TE: 1000/144 ms	NAA; Cr; Cho; NAA/Cr; Cho/Cr
Wootton-Gorges et al. [52]	2005	DKA: 25	12 ± 2.7	New onset (44%)	N/A	Periaqueductal region (A) and in the basal ganglia	Strength: 1.5 T TR/TE: 1500/144 ms	BHB; Lac; AcAc; NAA/Cr; PCr; Cho; acetone
Cameron et al. [51]	2004	DKA: 5 Non-DKA: 3	12.6 ± 3.2	New onset	N/A	Frontal cortex; occipital region	Strength: 1.5 T TR: 1500 ms TE: 30 ms	Tau; mI; glucose

Abbreviations: AcAc, acetoacetate; BHB, β-hydroxy butyrate; IAH, impaired awareness of hypoglycemia; N/A, none; NAH, normal awareness of hypoglycemia; tCho, total Cho; tCr: total Cr; TE, echo time; TR, repetition time.

### DKA

 DKA is one of the most devastating DM complications that leads to death in pediatric disease, which is usually complicated by cerebral edema [49]. Previous work has shown that there was neurone damage and loss during DKA progression. Wootton-Gorges et al. [50] detected brain metabolite levels during DKA treatment (2–12 h) and after recovery (>72 h) in the occipital grey matter, basal ganglia, and periaqueductal grey matter regions. They found that NAA/Cr ratios were significantly reduced in the basal ganglia during the DKA treatment period when compared with the recovery period (*P*=0.005). Similar trend was also found in the other regions tested. Cameron et al. [51] found that children with diabetes presented higher tau, mI, and glucose levels in frontal region when compared with children without DKA (*n*=8). Another study from Wootton-Gorges et al. [52] discovered a peak of β-hydroxy butyrate in the first 4 h of DKA treatment, while an acetone/acetoacetate peak was detected more than 4 h after treatment. Thus, data from studies regarding brain metabolite levels during DKA were inconsistent. Future studies using more standardized designs are required to elucidate this interaction.

### Hypoglycemia

Impaired awareness of hypoglycemia (IAH) affects nearly 25% of patients with T2DM [53]. However, evidence describing brain metabolite levels in patients with hypoglycemia is quite limited. A recent study from Wiegers et al. [54] tested a two-step hyperinsulinemic euglycemic (5 mmol/l) - hypoglycemic (2.8 mmol/l) glucose clamp on seven patients with IAH, seven patients with normal awareness of hypoglycemia (NAH) and seven healthy controls. The results showed that brain lac was decreased by 20% in the IAH patients when compared with both NAH patients and healthy controls (*P*<0.001). Lac may be reduced due to increased lac oxidation during hypoglycemia, which can result in neurone loss and impaired awareness. It still remains unclear whether levels of other metabolites change in the brain during hypoglycemic events.

## Brain metabolite alterations in diabetic patients with associated diseases

Besides retinopathy, kidney disease, and peripheral neuropathy, DM also causes damages to other end organs and induces DM-associated diseases. Details about the studies about brain metabolites in patients with DM-associated diseases are presented in Table 4.

**Table 4:** Clinical parameters of studies focussing on brain metabolite levels in diabetic patients with associated diseases (metabolic syndrome, cognitive dysfunction, depression and hypertension)

Study	Year	Number	Age (years)	DM duration (years)	HbA1c (%)	Testing area	Magnetic parameters	Identified brain metabolites
**Metabolic syndrome**								
Karczewska-Kupczewska et al. [60]	2013	High-IS: 8 Low-IS: 8	High-IS: 21 ± 2; Low-IS: 27 ± 3.6	N/A	N/A	Frontal cortex; thalamus; temporal region	Strength: 1.5 T TR: 1500 ms TE: 35 ms	NAA; Cho; Cr; mI; Glx; NAA/Cr; Cho/Cr; mI/Cr; Glx/Cr
Haley et al. [59]	2012	Control: 46 MetS: 19	Control: 50 ± 6.5 MetS: 51.9 ± 7	N/A	N/A	Occipitoparietal grey matter	Strength: 3.0 T TR: 3000 ms TE: 35 ms	NAA; Cho; Cr; mI; NAA/Cr; mI/Cr; Cho/Cr;Glu; Glu/Cr
Haley et al. [58]	2010	Control: 25 MetS: 13	Control: 51 ± 6 MetS: 48 ± 6	N/A	N/A	Occipitoparietal grey matter	Strength: 3.0 T TR: 3000 ms TE: 35 ms	NAA; Cho; Cr; NAA/Cr;Glu; NAA/Cho; Cho/Cr; mI/Cr
**Cognitive dysfunction**								
Wang et al. [65]	2015	NC: 266 DM: 188	NC: 60.5 ± 10.9 DM: 61 ± 9.1	6.4 ± 3.2	8.5 ± 2.2	Left hippocampus; the frontal lobe	Strength: 3.0 T TR: 2000 ms TE: 9.4 ms	NAA; Cho; Cr; mI
Tiehuis et al. [67]	2009	NC: 40 DM: 72	NC: 64.9 ± 5.4 DM: 66.5 ± 5.4	8.7 ± 5.6	6.8 ± 1.1	White matter	Strength: 1.5T TR/TE: 2000/144 ms	NAA; Cho; Cr; NAA/Cho; NAA/Cr; Cho/Cr
Lyoo et al. [66]	2009	NC: 38 DM: 123	NC: 30.8 ± 5.1 DM: 32.3 ± 4.4	19.9 ± 3.5	7.8 ± 1.4	Prefrontal region	Strength: 1.5 T TR: 1500 ms TE: 45 ms	Glx; NAA; glucose; Cr; Cho; mI
**Depression**								
Haroon et al. [69]	2009	Control: 19 DM: 20 DD: 18	Control: 54.5 ± 9.6 DM: 57.7 ± 7.9 DD: 56.9 ± 10.6	DM: 48.5 ± 11 DD: 47.2 ± 13	DM: 7.2 ± 1.1 DD: 7.3 ± 1.4	Bilateral prefrontal white matter	Strength: 1.5 T TR: 3000 ms TE: 30 ms	NAA; Cr; Cho; Ala; Asp; GABA; mI; Gln; Tau; glu; Lac; *scyllo*-inositol
Ajilore et al. [70]	2007	NC: 21 DM: 24 DD: 20	NC: 55.7 ± 9.9 DM: 58.1±9.2 DD: 56.6 ± 10.2	N/A	DM: 7.1 ± 1.1 DD: 7.5 ± 1.5	Dorsolateral white matter; subcortical nuclei region	Strength: 1.5 T TR: 3000 ms TE: 30 ms	NAA; Cr; Cho; NAA/Cr; Cho/Cr; mI; Glu; Gln
**Hypertension**								
Cao et al. [21]	2015	Control: 30 DHT: 33	Control: 59.7 ± 7.7; DHT: 62.8 ± 8.6	N/A	N/A	Frontal cortex; parietal white matter	Strength: 1.5 T TR: 1500 ms TE: 35 ms	NAA; Cho; Cr; NAA/Cr; Cho/Cr
Kario et al. [72]	2005	Control: 12 DHT: 20 HT: 20	Control: 69 ± 9.4 DHT: 69 ± 9.2 HT: 69 ± 9.2	N/A	N/A	White matter	Strength: 1.5 T TR: 1500 ms TE: 30 ms	NAA; Cho; Cr; NAA/Cr;

Abbreviations: DD, diabetic depression; DHT, diabetic hypertension; High-IS, high insulin sensitivity; HT, hypertension; IS, insulin sensitivity; MetS, metabolic syndrome; N/A, none; NC, normal control; TE, echo time; TR, repetition time.

### Metabolic syndrome

Metabolic syndrome (MetS) refers to a confluence of conditions including hyperglycemia, hypertension, and dyslipidemia [55], which has been shown to affect brain functions and memory [56,57]. A study by Haley et al. [58] on 13 patients with MetS and normal cognition compared with 25 healthy controls revealed significantly increased mI and glutamate levels in occipitoparietal grey matter regions. Additionally, mI/Cr (*P*=0.031) and Glu/Cr (*P*=0.035) ratios were increased. Similar results were found when the sample size was expanded to 65 patients [59]. Therefore, brain metabolic alterations may exist in patients with MetS, although participants did not exhibit cognitive impairments. And the MetS alterations are perhaps related to glutamate and mI metabolism. Another study of eight men with high insulin sensitivity (High-IS) and eight men with low-insulin sensitivity (Low-IS) using euglycemic–hyperinsulinemic clamp methodologies showed that, during insulin infusion, high-IS patients had increased NAA/Cr ratios in frontal lobe region, as well as both decreased Cho/Cr ratios in frontal cortex region and decreased mI/H_2_O ratio in temporal region [60]. However, these changes were not observed in low-IS group. These data suggested that brain metabolite differences occurred in response to insulin in patients with different insulin sensitivities. Insulin resistance is one of the most important factors in the development of DM. Although the 16 patients enrolled in the study presented with similar brain metabolic signatures at baseline, they presented with distinct brain metabolite levels and performances following insulin stimulation. The results also indicated that brain metabolic alterations could occur during pre-DM periods or in insulin-resistant patients. Future studies should focus on elucidating brain metabolite levels in prediabetic and insulin-resistant patients.

### Cognitive dysfunction

Significant alterations of brain metabolites induced by cognitive impairment or AD were reported in both human and animal models [14,61,62]. Numerous studies have revealed that prolonged hyperglycemia contributes to cognitive dysfunction and increases the risk of dementia [63,64]. Wang et al. [65] explored brain metabolite signature in the left hippocampus and frontal lobe of 188 T2DM patients and 266 healthy controls. To detect cognitive function, Mini-Mental State Examination (MMSE) and Montreal Cognitive Assessment (MoCA) were applied. DM patients had increased NAA, Cr, and mI levels in the left hippocampal region (*P*<0.05) along with lower MMSE scores, especially in aspects of attention, and language (*P*<0.05). Also, inverse correlations were found between MoCA score and both mI and Cr levels in the left hippocampal region of the T2DM patients (*P*<0.05). Lyoo et al. [66] studied with T1DM where diabetic patients had higher cerebral glucose and Glu-Gln-aminobutyric acid (Glx) levels (*P*=0.005), and lower cognitive performances in memory, executive functions, and psychomotor speeds (*P*=0.003). An inverse relationship was found between high Glx levels and poor cognitive performance (*P*<0.05). However, data from Tiehuis et al*.* [67] revealed that no differences were found in brain metabolites (NAA/Cr, Cho/Cr, and NAA/Cho ratios) when 72 T2DM patients and healthy controls were compared (*P*=0.13, 0.39, 0.89, respectively). No obvious correlations were discovered between cognitive performances and the above-listed brain metabolite ratios, but T2DM patients did exhibit poorer cognitive performances in memory and information processing speeds. The explanation for these data inconsistencies may result from the differences in study population. For example, Tiehuis et al. [67] study was performed on patients with an HbA_1_C of 6.8 ± 1%, while the HbA_1_C level in patients from Wang’s study [65] was 8.5 ± 2% [65]. As described above, brain metabolite levels could be likely influenced by the glycemic control and HbA_1_C levels in patients. Moreover, patients of different ethnicities could have metabolic signatures that reflect environmental and dietary variances. Therefore, more studies are required to explore the effects of DM on brain metabolite levels in patients with DM-induced cognitive impairments.

### Depression

DM and depression are linked by several etiologic factors [68]. Investigating altered brain metabolic signatures can elucidate mechanisms of the development of depression in DM patients. Haroon et al*.* [69] detected mI level in bilateral prefrontal white matter in depressed diabetic patients (*n*=20). The results showed that mI and mI/Cr ratio in right frontal white matter were increased in DM patients when compared with healthy controls (*P*<0.05). Correlative analyses identified an inverse relationship between mI levels and recall as well as recognition subtests (*P*<0.05). Similar mI trend was reported by Ajilore et al. [70] in 20 diabetic patients with depression, 24 diabetic patients, and 21 healthy controls. In addition to increased mI levels, Glu and Gln levels were decreased significantly in subcortical regions of depressed diabetic patients when compared with DM patients and healthy controls (*P*<0.001). Thus, these findings on mI, Glx levels might provide insights into the pathological underpinnings of the development of depression in DM patients.

### Hypertension

Based on the data from World Health Organization (WHO), nearly 60% of patients with T2DM have concomitant hypertension, so brain metabolite alterations may be more pronounced in such patients [71]. Cao et al. [21] investigated brain metabolites in the frontal cortex and parietal white matter of 33 patients with diabetic hypertension (DHT) and 30 healthy controls. The results showed that both NAA/Cr and Cho/Cr ratios were significantly decreased in the frontal cortex regions (*P*<0.01). Similarly, Kario et al*.* [72] showed that only NAA/Cr ratio was reduced in the parietal white matter when compared with healthy control patients *(P*<0.001). The authors concluded that DHT could cause metabolic disorders in the brain in a region-specific pattern, and NAA/Cr and Cho/Cr ratios might be biomarkers indicative of this damage. Due to limited evidence investigating DHT patients and small sample sizes in the previous studies, it is doubtful that measurements of NAA/Cr and Cho/Cr ratios were powerful enough to serve as reliable biomarkers for DHT patients.

## The underlying mechanism of the altered metabolic molecules and their effects on brain functions

**Figure 1:**
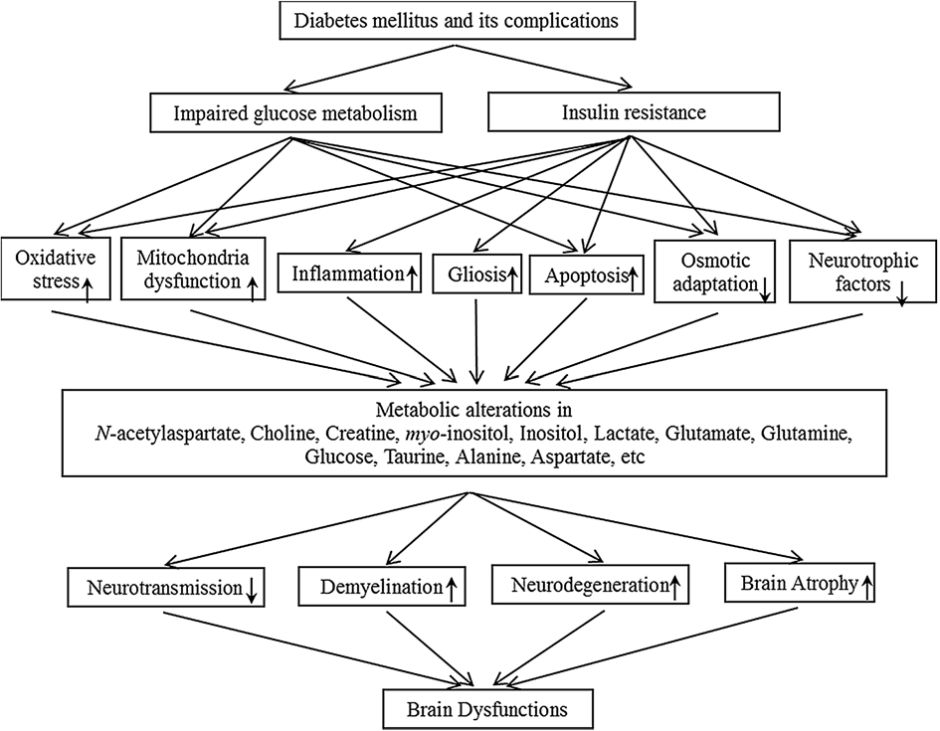
The possible underlying mechanism of diabetes-induced brain dysfunctions.

Because ^1^H MRS technology can detect the altered metabolic molecules non-invasively, it is widely used for early diagnosis, and biomarker exploration for neurological diseases, including diabetes-induced brain dysfunction [73]. The alterations of metabolic molecules examined can convey much information about the underlying mechanism of diabetes-induced brain dysfunction, and the accumulations of these molecules can also aggravate the brain dysfunctions, resulting in a vicious circle. Studies have shown that when diabetes and its complications happen, glucose metabolism is affected and insulin resistance will happen [74,75]. Impaired insulin signaling will influence energy metabolism, inducing mitochondria dysfunction, enhanced oxidative stress, ROS accumulation, increased neuro-inflammation, apoptosis, gliosis, and decreased neurotrophic factors [76,77]. Long-term hyperglycemia can cause impaired osmotic adaption, apoptosis, and so on (Figure 1). To reflect these pathological processes, the concentrations of metabolic molecules in brain will change. For specifically, NAA is frequently distributed in the brain. It is mainly located in neurones, especially in mitochondria. Thus, when neuronal density, function, or viability is affected, NAA level will change. And the change of NAA conveys the information about neurone loss [78,79]. Cho is involved in membrane metabolism, when the concentration of Cho changes, it means the cellular membrane is damaged [80]. Cr takes part in the energy metabolism, and increased Cr level means increased oxidative stress, ROS and mitochondria dysfunctions in both neurones and glial cells [81]. As for *myo*-inositol, it has been known as the marker of gliosis. When insulin resistance happens, inflammation will be enhanced, inducing the process of gliosis of glial cells [82]. And all above metabolic alterations will lead to disturbed neurotransmissions, accelerated the neurodegeneration and demyelination as well as induce brain atrophy. Finally, brain functions will be influenced in a large scale. Different stages of diabetes and different diabetic complications might present inconsistent levels of metabolic molecules, which might help to differentiate or diagnose the diabetic complications earlier. However, the exploration of the underlying mechanism of these metabolic molecules is at the initial stage, future study should validate above findings in larger-scale clinical studies.

## Effects of diabetic treatment on brain metabolites

Since DM and its complications have effects on brain metabolites, application of diabetic treatments may restore brain metabolism and provide insights into treatments of other brain disorders. Most available studies have focussed on detecting brain metabolite levels in DM patients that are participating in physical exercise. Nagothu et al. [83] investigated 68 patients with T2DM, half of the patients did 6 months of yoga, while the rest half of the patients were not assigned a task. After 6 months, NAA levels were increased (*P*<0.02) and mI levels were decreased (*P*<0.01) in the right dorsolateral frontal lobe regions of the exercising patients when compared with the controls, suggesting that long-term exercise regimens restored altered brain metabolite levels in diabetic patients. However, Santhakumari et al. [84] reported that yoga had no effect on brain NAA and mI levels based on an analysis of five DM patients. Nevertheless, they found that yoga ameliorated memory decline in diabetic patients (*P*=0.001). Due to the small sample size in the latter study, it is difficult to conclude that exercise had a meaningful influence on brain metabolism, even in patients who experienced a significantly decreased HbA_1_C (*P*=0.03). Thus, large-scale studies are required to elucidate this interaction. Moreover, future studies could explore the role of different DM treatments on brain metabolite levels to provide alternative explanations for the memory declines and brain dysfunctions observed in DM patients.

## Conclusion

In the present review, associations between DM and its complications on brain metabolite levels were discussed. Cerebral metabolite levels including NAA/Cr, mI/Cr, Cho, Glu, and Gln were significantly altered in patients with DM when compared with healthy controls, suggesting that energy metabolism, neurotransmission, and lipid membrane metabolism were impaired in patients during DM progression. Monitoring changes in brain metabolites could be used as non-invasive biomarkers for progression of DM and development of related complications. Additionally, MRS data illustrating brain metabolite levels can elucidate and monitor the effects of DM treatment. Diabetic treatments such as exercise might restore altered brain metabolite levels and provide beneficial effects on cognition in DM patients. Future studies should validate the above findings in larger population samples and further uncover the mechanistic underpinnings of DM-induced brain damages.

## Abbreviations

AD: Alzheimer’s disease
Cho: choline
Cr: creatine
DHT: diabetic hypertension
DKA: diabetic ketoacidosis
DKD: diabetic kidney disease
DM: diabetes mellitus
DPN: peripheral neuropathy
DR: diabetic retinopathy
ESRD: end-stage renal disease
GABA: γ-aminobutyric acid
Glx: glutamate + glutamine
HbA_1_C: glycosylated hemoglobin
High-IS: high insulin sensitivity
IGT: impaired glucose tolerance
IAH: impaired awareness of hypoglycemia
Lac: lactate
Low-IS: low-insulin sensitivity
MetS: metabolic syndrome
mI: *myo*-inositol
MMSE: mini-mental state examination
MoCA: Montreal Cognitive Assessment
MRS: magnetic resonance spectroscopy
NAA: *N*-acetylaspartate
NAH: normal awareness of hypoglycemia
NPDR: non-proliferative DR
PDR: proliferative DR
T1DM: type 1 DM
T2DM: type 2 DM
UAER: urinary albumin excretion rate
